# Advice to Young Doctors

**Published:** 1886-04

**Authors:** A. G. Haygood

**Affiliations:** Oxford, Ga.


### ADVICE TO YOUNG DOCTORS.

BY A. G. HAYGOOD, D. D., LL. D., OXFORD, GA.

ANNUAL ADDRESS TO THE GRADUATING CLASS OF THE ATLANTA MEDICAL COLLEGE DELIVERED IN DE GIVE'S OPERA HOUSE MARCH 1, 1886.

YOUNG GENTLEMEN—As to my relation to the proprieties on this occasion, one thing at least is clear to me—my address should be a short one, and in this respect I hope to please you.

First of all, I wish to say the profession you have chosen, although it is burdened with incompetent men, is not, in any sense that should in the least discourage a real man, over-crowded. Medicine is not at all peculiar as to the matter I ask you to consider. Look where you will, the masters are few. Law, theology, journalism, school-teaching, furnish ready and abundant illustrations. So do the mechanic arts; of all pursuits farming, perhaps, furnishes the most striking illustrations. No doubt there are too many men who have diplomas from medical colleges, just as there are too many who have certificates of admission to the bar, and as there are too many who have some sort of license to preach. You do not need that one should prove that diplomas no more make doctors than certificates make lawyers or ordination papers make preachers. Yet there are not a few who do not seem to understand these truisms. These are they who bring reproach on their professions, to say nothing of the harm they do the unlucky ones who fall into their hands.

For the needs of the people, there are not enough doctors who are doctors. I mean this: men of such sense, science, skill and fidelity that a father, whose child may die under their care, may have at least this consolation, his child had something like the best that medicine could do for it.

Such an assurance makes a great deal of difference to bereaved people; it ought to be everything to the physician. Four children have been buried from my house; in one case the attending physician, the best in reach at the time, was candid enough to say a little while before the child died, that he did not know how to treat sick children. In two other cases I had reason to believe that the children had competent attention; in one case there was reason to believe that, humanly speaking, a blunder in diagnosis, followed by a blunder in treatment, killed the child. Toward the physician in this case I had grace to feel kindly, for it was evident that he did the best he knew. He never realized his incompetence; he did not know enough. Had he known more he would have studied his business more thoroughly, for he was not lacking in conscience.

The majority of people are ill-informed about all important things; perhaps they know as little of disease and medicine as anything else that concerns them. The ignorance of the people gives, in every department of business, quacks and impostors the best chance for temporary success. That is, as a rule, they will attract most attention and make most money for a time. But in the long-run—and it is not worth while to philosophize about short-runs—it may well be doubted whether many truly capable doctors are long without patients. If you will tell me of a thoroughly furnished physician, who, after fair trial in patient waiting and honest effort, in a reasonably sickly community, is long without practice, I will tell you of a man who has in himself some quality that explains his failure. He may be learned in books, but without common sense; skilled in the use of instruments, but without good judgment as to the occasions for using them; accurate in diagnosis, but unreliable in prognosis and confused or timid in prescription. Some fail because they are ill-mannered, and not a few because they are indolent, and some because they are intemperate in the use of stimulants. In a word, the failure has an explanation outside the doctrine of luck, and that does not prove that there are more good doctors than the people need.

The old saw, "there is room enough at the top," has a great deal of truth in it. I suppose it will always be a true saying; but it would be unwise to conclude that the crowds that are at the bottom in medicine are failures there in any sense that is peculiar to that profession. As to the majority, it is probable that they do as well there as they would anywhere, with this difference, that failure in the other professions is not so risky to other people. In finding fault with our own or commenting on other people's business, and what we consider failure, we sometimes forget that, in the accepted sense of the word, success is not achieved by the majority in any of the pursuits of men.

A common trouble with us all is that we fail in our business because we think little of it. No man truly succeeds in any calling who has a poor opinion of it. No man has a good opinion of his business who only cares for it for the living that is in it. No man has the best opinion of his business who uses it only to make money out of it. No man can have the best conception of his business who does not esteem it for itself and for its usefulness, and the higher we go—if higher and lower are proper terms to use in considering the different honorable and useful walks of life—the more clearly will it appear that he who only esteems his business for the living or the money that is in it must, if judged by any high standard, be a failure.

No man, in any business fit for a man to follow, can do his best unless he idealizes his business and makes it truly his calling. He must form for himself the largest conception he can of his calling—of what it ought to be. Then, if he be a true man, he will work towards his ideal, not merely for a living or for gain. If Marion Sims had thought of medicine only as a means of making a living, he might have made a living—hardly more—but he would not have brought mankind under obligation to him. It was his ideal—his high conception of what a doctor ought to be, and therefore could be—that lifted him so high that the civilized world honors him as it honors few men of our time. Toward the realization of that conception of his work and office he worked faithfully, and I may say lovingly to his dying day, ever seeking to learn more and to do better work. His noblest contributions to medicine came, not out of palaces, but hovels; not when he was getting the largest fees, but when he was in love with his work and devoted to it, trying hardest to be what a doctor ought to be without so much as thinking of money.

Many circumstances, not needful to mention now, have made me care for medicine and doctors. In childhood I "suffered many things of many physicians," and have hardly needed them since. This, perhaps, is enough to say. Next to my own the doctors' work has most interested me. I will not undertake to advise; if I could encourage you to-night, I would be glad.

Most of you will be village and country doctors, with the half-formed but conscious purpose some day to get to the city, where it is believed the great triumphs are to be won.

Let us for a moment consider the case of the village and country doctor. In the first place, in our section, he is in the majority. In the second place, he, above all doctors, needs to be most thoroughly furnished, for he must depend most on himself. Country practice places the doctor at many disadvantages. His cases are as difficult as humanity can afford and his opportunities are least. He may need counsel and find himself alone, or what is worse, dependent for consultation upon an ignoramus or a bigot. He may need the best skill of the best druggist, and find it necessary to be pharmacist as well as physician. He may be confronted with the most complicated case known to surgery, and lack instruments and appliances. He needs to know all things and to have all things. Dr. Alexander told his students in theology: "When you go to the city to preach, wear your best coat; when you go to the country, take your best sermon."

For a different reason, it may be, but for a good one, I may say to your faculty, send your best men to the country.

I have known village and country doctors like those I am going to describe: His office an ill-kept, dusty lounging place. While waiting for patients he loafs at the village store or cross-roads post-office, playing drafts (he rarely rises to the dignity of chess) or whittling, while telling or hearing old stories or new reports. He is bird-hunter, trader, politician, store-keeper, farmer, loafer by turns. You also have seen him. What can he do? If you go to the country, do your best. . . . .

What does your diploma signify? Mostly this: Your preceptors think you are a suitable man to become hereafter a doctor. In some cases they may be mistaken, and the failure that is sure to follow will not be due to the conditions of business in an overcrowded profession, but to a misjudgment in the choice of a profession. It is easy to make a mistake like this. Some choose medicine because it is respectable; some because they thought it an easier way to make a living than some other way that was suggested at the time of choosing; some because parents and friends persuaded them. You will soon find out, if you try, whether you are "truly called to this office and ministry." If you are you will love and honor your business, and study, as never heretofore, to make yourselves approved in it.

A great deal of nonsense appears in the papers and elsewhere about practical education. The literary college is sneered at. So is the law-school, the medical college and the seminary. Nothing is easier than to make a flippant criticism. These self-satisfied critics seem not to know that practical education must, for the most part, come by practice.

A man cannot learn to be an athlete by any amount of study or observation of gymnastic feats. He must himself perform them before he can know how to do them, to say nothing of skill in doing them. It is so everywhere.

Young doctors, there is no help for it; you must practice on us of the laity before you can become really practical physicians. We will protect ourselves as long as we can. While the "old Dr." lives and is available we will, if we get sick, send for him. When we can do no better, we will send for you. Don't worry at this; it must be so; it is our only defense; you will have your revenge soon enough. The old Dr. will die some day, or he will be too busy to come; something will befall us; the attack will be as sudden as severe; we must have your help or none. We may have laughed at you, and in our folly may have vowed that we would not send for you to treat a sick dog, but for all these follies of ours we will with desperate resolution send for you, trusting to a merciful Providence to help us through. (And we will trust Providence the more readily when we see even the young doctor, as badly scared as his patient, at the bedside). If we die you can explain it; if we get well we will sound your praises, even if we are slow in paying your fee.

If you kill a good many of us while really learning the practical part of your business, don't take it too much to heart, or throw your sign in the well, as Dr. Sims did after killing two or three patients.

The doctor is not the only man certain to make failures at the start. The young lawyer loses first cases also before he learns how to save the precious necks of those who ought to be hung. Preachers are in like case. We pass our blundering and humiliating novitiate, and disgust, with our platitudes and noise, many congregations before we are useful to any.

What young doctors are to be blamed for is, not that they kill some of their first patients, but that they do not consider that they run the risk of killing them all, and by hard study and earnest effort at least lessen the dangers to which they are exposed.

It is to be suspected that not a few physicians have only one year's experience, although they have been practicing for many years. At the end of twenty years, they do just as they did the first year—they have learned nothing beyond their first lessons. There is perhaps no work so simple that its possibilities can be exhausted by the first efforts. If a man is only digging post-holes, he will find that he needs more than mere muscular force in order to do the work well and rapidly. In such a work as the physician's, so varied, so complicated, so conditioned by hundreds and thousands of influences, there is never a time to cease learning; never a time when opinions do not need revision; when new facts are unimportant. If there is one man in the world more than another who must keep up with discovery, it is the physician; if there is one man more than another who should be tolerant of differing opinion, it is the physician; if there is one more than another whose mental attitude should be free from bias and prejudice, it is the physician.

But to keep my promise of brevity, it is time to look to the conclusion. For your meditation hereafter, if you think them worthy of consideration, I suggest one or two points that I will not discuss:

1. It seems to me, from the little I have learned of medical history, that the noblest achievements have been won by those who were seeking rather to prevent than to cure disease. Many illustrations might be offered, but you know them already. More than ever in the future, it should be true that medicine makes progress in seeking to prevent disease, for all the sciences now work in partnership, and medicine never had such resources for finding out the nature of disease, the conditions of its origination or dissemination as well as the means of arresting it.

Perhaps, after all, the Chinese have the right view—to pay the doctor while in health and to stop his fees with the coming of disease.

The medical profession is singular in this: the ideal doctor is one whose complete success would make an end of his business, for his true professional instinct is to prevent the maladies which make his services necessary.

2. While the doctor in ordinary practice should study to be ready to do his part in any sort of practice that may come to him, it seems to me that he should select for special study and observation some particular thing—a part of the body, a disease, a remedy—something that he may make notes on for the benefit of his profession. It may be, comparatively speaking, a very little thing, but it may also be that it is the one thing needing to be understood. A very plain, unpretentious man may, by using his odd hours and moments for some special study, discover in a given department the one thing lacking. It is surprising how many great discoveries seem to have been made by accident; but the discoverers had at least the habit of observation. If among twenty country doctors—I speak of these because there are so many of them and so much depends upon them—ten only would, besides general study for general practice, specialize in their studies and observations some one thing, they would hardly fail among them to make important discoveries. And when the observing physician has found out a secret that every human being has interest in, let him proclaim it to the world. He may make more money by locking his discovery in a government patent, but the true doctor does not come into the world to make money, but to prevent and cure disease.

Suppose--which, thank God, can't come to pass—that a preacher should discover something about human salvation unknown to others and keep it to himself—for his own profit? What would you think of him? He would be turned out of the church.

3. Perhaps the navigators will find no more continents, unless they could also find the poles. Whether medical science has ever found all the continents yet, a layman is not prepared to discuss. But the most valuable achievements are not simply in finding a continent, but in exploring and settling it. The Northmen, it seems, found North America long before Columbus sailed, but their discovery issued in no results of moment. We have hardly as yet more than begun to find out what this continent contains for the welfare of man. One does not need to be learned in medicine to reach the conclusion that the greatest discoveries are before and not behind us. Any day nature may yield to science a new secret; any day a discovery may be announced that will bless while it astonishes the world.

Do not, young gentlemen, enter upon your profession with the depressing idea that there are not new worlds for you to conquer. Theoretically our finite condition limits progress; practically there are no limits, for the race has not yet discovered them. One need not repine that the seas are not absolutely boundless, while he is yet in mid-ocean with no prospect of ever reaching land. Heretofore the men of the South have not been lacking in important—I may say immortal—contributions to medical science. Hereafter they should do ten times more, for their opportunities are tenfold greater, and do you, young men of the graduating class, be sure to do your part.

